# Long‐term cost‐effectiveness of invasive urodynamic studies for overactive bladder in women

**DOI:** 10.1111/bju.16703

**Published:** 2025-04-19

**Authors:** Helen Bell‐Gorrod, Praveen Thokala, Suzanne Breeman, David Cooper, Graeme MacLennan, Mohamed Abdel‐Fattah, Simon Dixon

**Affiliations:** ^1^ School of Medicine and Population Health University of Sheffield Sheffield UK; ^2^ Health Services research Unit University of Aberdeen Aberdeen UK; ^3^ Aberdeen Centre for Women's Health Research University of Aberdeen Aberdeen UK

**Keywords:** cost‐effectiveness, urodynamic testing, overactive bladder, treatment, randomised controlled trial

## Abstract

**Objectives:**

To estimate the cost‐effectiveness of using invasive urodynamic studies (UDS) in the management of women with refractory overactive bladder (OAB) symptoms using the results of the FUTURE trial.

**Patients and Methods:**

The FUTURE study is the largest randomised controlled trial evaluating the clinical effectiveness of UDS with comprehensive clinical assessment (CCA) in this patient population compared to CCA only. We developed an economic model that replicates the 24‐month results of FUTURE, then models the lifetime costs and quality‐adjusted life‐years (QALYs) using long‐term studies of treatment outcomes.

**Results:**

Over the patient cohort's lifetime the UDS plus CCA group is £1380 more costly and is associated with 0.002 fewer QALYs than the CCA only group, with only a 23.4% chance of being cost‐effective at £20 000 per QALY gained. The sensitivity analysis shows that the results are robust to all changes except for the use of parameters based on the complete case analysis of the FUTURE trial. For the subgroup of patients with an initial diagnosis of mixed urinary incontinence, the UDS group gains more QALYs than the CCA group, albeit at a higher cost. The incremental cost‐effectiveness ratio for UDS is £26 462, with a probability of being cost‐effective of 45.3% at £20 000 per QALY gained and 53.8% at £30 000 per QALY gained.

**Conclusion:**

The use of UDS in women with a diagnosis of OAB and whose condition is refractory to initial medical and conservative treatments is unlikely to be cost‐effective when examined from a UK perspective and with a lifetime horizon. Despite having access to the FUTURE study data, the parameterisation of the model is limited by the current evidence base. An ongoing long‐term follow‐up study will help reduce these uncertainties.

**Abbreviations**
CCA: comprehensive clinical assessment
CEAC: cost‐effectiveness acceptability curve
CEP: cost‐effectiveness plane
COVID‐19: coronavirus disease 2019
BoNT‐A: botulinum toxin A injection
EQ‐5D‐5L: EuroQoL five Dimensions five Levels
GLM: generalised linear model
HEAP: Health Economics Analysis Plan
NICE: National Institute for Health and Care Excellence
(R‐)OAB: (refractory) overactive bladder
PTNS: percutaneous tibial nerve stimulation
QALY: quality‐adjusted life‐year
RCT: randomised controlled trial
SNM: sacral neuromodulation
UDS: urodynamic studies
(M)(S)UI: (mixed) (stress) urinary incontinence

## Introduction

Overactive bladder (OAB) is a symptom complex of urinary urgency, with or without urgency urinary incontinence (UI), usually with increased daytime frequency and nocturia, and with no proven infection or other obvious pathology. OAB has negative impact on women's social, physical, and psychological wellbeing, and negative effects on working women's productivity. In a study of women referred to a urodynamic clinic, 53% women reported that employment was affected, 60% avoided leaving home, and 40% reported avoiding sexual activity [1].

The EPIC study estimated the prevalence of OAB to be 11.8% with further research showing that it rises with age and individuals from African backgrounds have higher rates [2, 3]. First‐line treatments for OAB include lifestyle changes, pelvic floor muscle training and medical treatment such as anti‐cholinergic or β‐adrenergic receptor agonists. However, up to 40% of women with OAB will not show improvement, i.e., refractory OAB (R‐OAB) [4]. In women with R‐OAB, the National Institute for Health and Care Excellence (NICE) recommends urodynamic studies (UDS) assessment to confirm the possible underlying diagnosis of detrusor overactivity prior to proceeding for treatments such as an injection of botulinum toxin type A (BoNT‐A) into the bladder wall or sacral neuromodulation (SNM) as management for R‐OAB [5].

The FUTURE Study is the largest randomised controlled trial (RCT) evaluating the clinical and cost‐effectiveness of UDS in the management pathway of women with idiopathic R‐OAB or urgency predominant mixed UI (MUI). Our results confirm that the participant‐reported success rates following treatments in women who underwent UDS plus comprehensive clinical assessment (CCA) were not superior to those who underwent CCA‐only (odds ratio 1.12, 95% CI 0.73–1.74; *P* = 0.60) [6].

Whilst the main clinical analyses of FUTURE show that the use of UDS does not improve participant‐reported success rates compared to CCA, an economic analysis is still considered important as small differences at 24 months could lead to important cumulative cost and outcome differences of the patients’ lifetimes. This study reports on the cost‐effectiveness of UDS as described by the FUTURE study, through the development of a decision analytical model by utilising external data relating to the long‐term effectiveness of subsequent treatments.

## Patients and Methods

The model represents those patients recruited to the FUTURE study, specifically, women aged ≥18 years with R‐OAB or urgency predominant MUI, with failed conservative management and being considered for invasive treatment. The economic evaluation took the form of a cost‐effectiveness analysis with outcomes measured by quality‐adjusted life‐years (QALYs). The primary analysis was based on modelling the lifetime costs and QALYs of the patients included within FUTURE. The modelling methods are in line with those of the NICE [7], whilst the trial‐based analysis follows international methodological guidelines [8]. In line with the NICE methods, the evaluation takes a NHS perspective. Methods were pre‐specified in the study protocol [9] and health economics analysis plan (Appendix S1), with all deviations reported in the Discussion.

### Model Structure

**Fig. 1:**
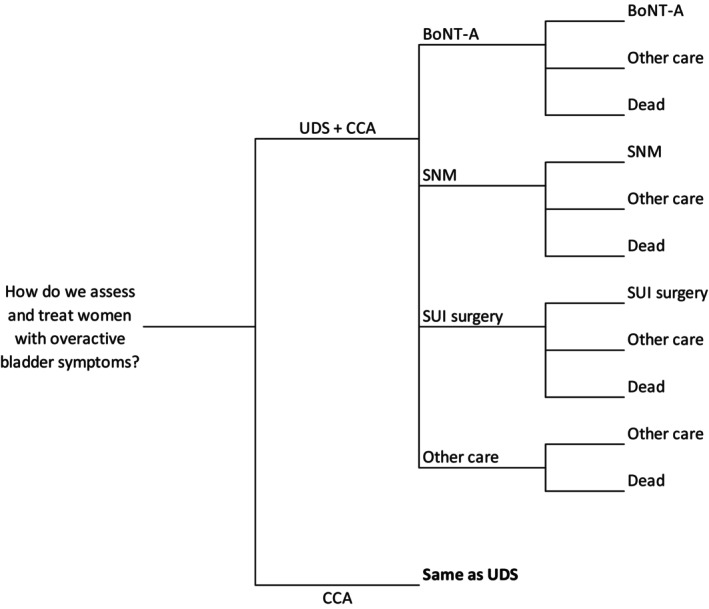
Model structure.

The model‐based analysis used a hybrid model structure with a decision tree describing short‐term events and a Markov process describing long‐term events and is shown in Fig. 1. The model describes a simplified set of pathways following randomisation, starting with an initial treatment decision (as observed in FUTURE), to which time‐dependent success rates are applied (from the literature) and, finally, the initiation of other treatments. The structure of the model is based on NICE treatment pathways and previous economic evaluations of associated treatments [10, 11, 12, 13, 14].

The model takes the estimated total costs and QALYs at 24 months directly from the within‐trial analysis (see Cost and QALY inputs). Successfully treated participants remain on that treatment, with treatment failures moving to ‘other care’. The proportion of participants receiving BoNT‐A, SNM, or having received surgery for stress UI (SUI), or receiving ‘other care’ at 24 months were taken from FUTURE. Transitions beyond 24 months were based on a review of longitudinal studies and previous models (see Probabilities, below).

### Probabilities

Searches for the long‐term outcomes of treatments following UDS or CCA for women with R‐OAB uncovered a systematic review, which included four studies [15]. These were inadequate for our purposes as they had a maximum follow‐up of only 36 months and did not disaggregate outcomes by treatment. Consequently, we undertook our own searches for cohort studies of women with R‐OAB who received either BoNT‐A, SNM or surgery for SUI. Eight studies were identified as relevant and these were used to generate success rates for the three treatments (Table 1), together with revision rates and time to revision for SNM, and interval between treatments for BoNT‐A (see notes to Table 1) [16, 17, 18, 19, 20, 21, 22, 23]. All‐cause mortality was estimated annually using population life tables based on the mean age of the FUTURE participants of 60 years [24].

**Table 1:** Transition probabilities associated with long‐term success rates.

Time, years	SNM success rates*	BoNT‐A, % remaining on treatment^†^	SUI success rates, %^‡^
1	77.1	64	84.0
2	75.6	51	78.4
3	74	43	74.3
4	72.4	38	71.1
5	70.9	38	68.4
6	69.3	38	66.0
7	67.7	38	63.9
8	66.1	38	62.1
9	64.6	38	60.3
10	63	38	58.8

* Based on studies by Kaaki and Gupta [16] and Ismail et al. [17], the associated standard error being based on a sample size of 55 patients [16].

^†^ Based on Mohee et al. [18], which reports on a cohort of 137 patients.

^‡^ Based on a curve fitted to 1‐ and 2‐year probabilities relating to mid‐urethral slings, from a systematic review reported by Brazzelli et al. [19] of 84% and 78.4%, respectively. The estimated curve is a Weibull with scale and shape parameters of 0.174 and 0.485, respectively (and standard errors of 0.0348 and 0.12125).

### Cost and QALY Inputs

Participant level data were collected in FUTURE for the trial interventions (UDS plus CCA vs CCA‐only), plus subsequent treatments, investigations and other health service contacts. Data were collected at 6 and 15 months after randomisation for all patients, and at the 24‐month follow‐up for patients who had their treatment delayed due to the coronavirus disease 2019 (COVID‐19) pandemic. Data for hospital‐based care were collected from medical records, with all other items being collected via participant questionnaires. A full list of resource use items is given in Appendix A: Table A1.

Unit costs are at 2020/2021 price levels and are based on national estimates (Appendix A: Table A1). QALYs were calculated using the EuroQoL five Dimensions five Levels (EQ‐5D‐5L) values measured at baseline, 6, 15 and 24 months after randomisation, where appropriate. The EQ‐5D‐5L responses were valued using the ‘cross‐walk’ tariff recommended by NICE at the time of protocol development. Both costs and outcomes were discounted at 3.5% per annum.

The within trial analysis, which provides the 2‐year cost and QALY parameters for the model, was based on regression analysis of individual patient data. Regression models were fitted based on an assessment of the distributions for the cost and QALY data. The cost regression used a generalised linear model (GLM) with a gamma family and identify link, and the QALY regression used a GLM with Gaussian family and identify link. Regressions used the following covariates that were in line with those for the clinical analysis; pre‐randomisation diagnosis (OAB vs MUI), age, age squared, number of deliveries, urgency perception and study centre dummy variables). Follow‐up time and follow‐up time squared were used as additional covariates as both costs and QALYs are fundamentally linked to length of follow‐up. Additionally, the QALY regression included baseline utility score as a covariate. The validity of imputing missing data was assessed based on an assessment of patterns and predictors of missingness. Multiple imputation was used to account for missing data using age, OAB dummy, 24‐month follow‐up (yes/no), number of deliveries and urgency perception as predictors. The cost and QALY estimates used within the model and for the secondary (within‐trial) analysis, are the predicted 24‐month values based on these regressions (Table 2). As such, the costs and QALYs for patients with only 15 months of follow‐up are statistically adjusted based on the data of those patients with 24 months of follow‐up (having taken into account the aforementioned covariates).

**Table 2:** Model inputs.*

Parameter		Time point(s), months	Mean
**Costs at 24 months (discounted), £**			
UDS + CCA		Up to 24	3907.33
CCA		Up to 24	3444.78
**QALYs at 24 months (discounted)**			
UDS + CCA		Up to 24	1.315
CCA		Up to 24	1.304
**Last treatment at 24 months, %**			
UDS + CCA	BoNT‐A	At 24	49.27
UDS + CCA	SNM	At 24	1.82
UDS + CCA	SUI surgery	At 24	2.55
UDS + CCA	Other	At 24	46.36
CCA	BoNT‐A	At 24	61.93
CCA	SNM	At 24	1.09
CCA	SUI surgery	At 24	0.73
CCA	Other	At 24	36.25
**Utilities at 24 months for**			
	BoNT‐A	After 24	0.632
	SNM	After 24	0.599
	SUI surgery	After 24	0.643
	Other	After 24	0.612
**Unit/annual costs (undiscounted), £**			
	BoNT‐A (applied to re‐treatment)	After 24	463.75
	SNM (applied to revisions)	After 24	1614.97
	SUI surgery	After 24	0
	Other treatments, e.g., PTNS, urethral bulking	After 24	1723.31

* Data relating to the associate distributions around the means used for the probabilistic sensitivity analysis are shown in Appendix B: Table B1.

The costs for BoNT‐A, SNM replacement and SNM removals within the model are the same as those for the within trial analysis. The cost for ‘other care’ is estimated from FUTURE using the observed cost of participants who had not received any of the alternative treatments up to the end of their trial follow‐up (Table 2). ‘Other care’ includes hospital visits, incontinence pads, catheters and related medications, including antibiotics for UTIs.

### Analysis

The primary analysis is the model‐based analysis using a lifetime horizon, with the secondary analysis being based on the 24‐month within‐trial analysis. The principal outputs are the mean incremental costs and QALYs, the incremental cost‐effectiveness ratios, together with their associated cost‐effectiveness acceptability curves (CEACs) in relation to a threshold of £20 000 per QALY gained.

Deterministic sensitivity analyses relating to the primary analysis focused on parameter‐based uncertainties; a reduction in the extrapolation period to 5 years and equal utilities for all women receiving BoNT‐A, SNM or surgery for SUI. The former was undertaken in recognition of the weaknesses found in the evidence base relating to long‐term effectiveness, whilst the latter was undertaken in recognition of the fact that patient numbers receiving SNM or surgery for SUI are very small at 24 months (1.82% vs 2.55%), and therefore, observed utility differences could be misleading.

Deterministic sensitivity analyses for the trial‐based analysis were undertaken in relation to methodological uncertainties. They assessed the impact of a complete case analysis, use of an alternative cost for UDS produced by bottom‐up costing [25], use of an alternative utility tariff for the EQ‐5D‐5L [26], and the inclusion of additional predictors within the multiple imputation.

Probabilistic sensitivity analyses were undertaken using 1000 samples from the associated distributions, which capture the degree of sampling uncertainty of the underlying data source (Appendix B: Table B1). Results were plotted on the cost‐effectiveness plane (CEP) and used to generate CEACs. The CEP shows how incremental costs and QALYs vary with different parameters samples, whilst the CEAC uses the same information to determine the probability that UDS + CCA is cost‐effective at different funding thresholds.

A sub‐group analysis was undertaken for the primary and secondary analyses based on pre‐randomisation diagnosis (OAB vs MUI). Within FUTURE, the responsible clinician determined the type of UI after taking a clinical history and undertaking a physical examination. Estimation of 24‐month costs and QALYs for the sub‐groups were undertaken by the addition of an interaction term to the aforementioned regression models.

## Results

**Fig. 2:**
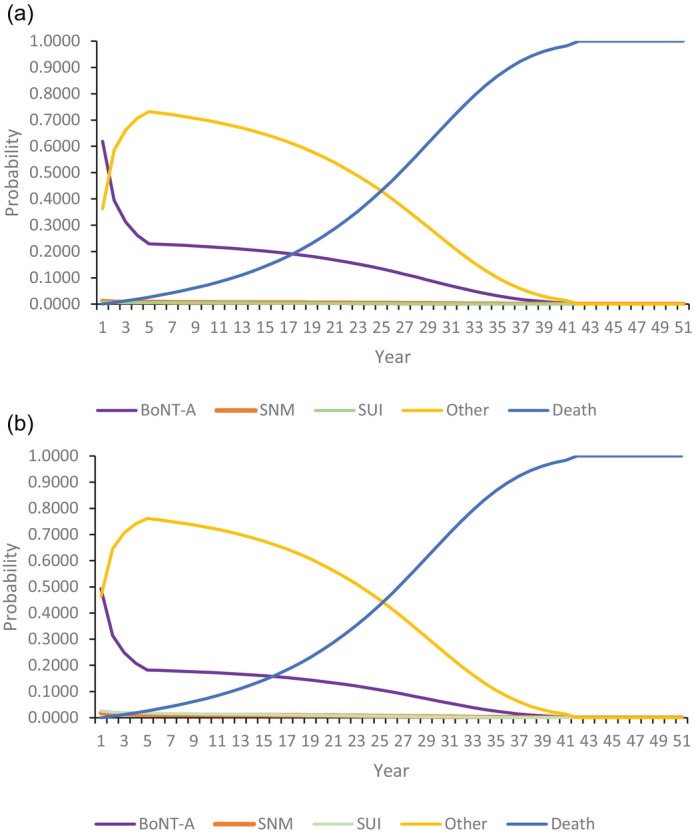
Markov traces. (**a**) CCA. (**b**) UDS plus CCA.

The projected movement of women between treatments beyond the end of the trial is shown in the Markov traces in Fig. 2. The four lines show the proportion of women, by year, who are assigned the costs and QALYs relating to BoNT‐A, SNM, SUI, ‘other care’, or death. This shows women moving from BoNT‐A and SNM onto ‘other care’ for the first 5 years after the trial. Beyond that point, the increasing mortality seen with ageing in the general population becomes the dominant factor (noting that the starting age for the modelled cohort was 60 years).

**Fig. 3:**
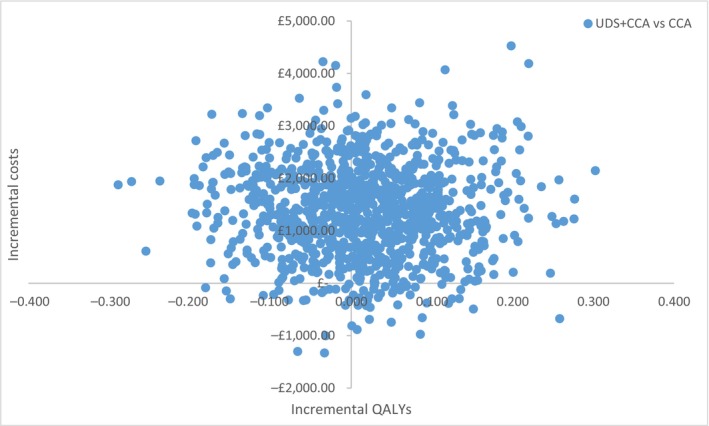
Cost‐effectiveness plane of UDS and CCA vs CCA‐only of the lifetime of women.

**Fig. C1:**
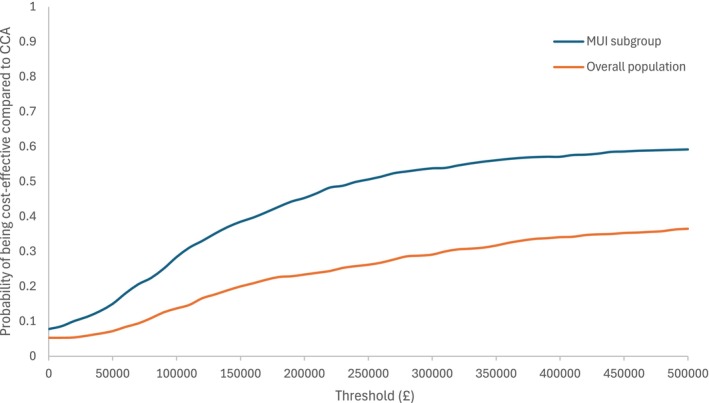
Cost‐effectiveness acceptability curve of UDS + CCA vs CCA‐only of the lifetime of women.

When costs and utilities are applied to these transitions, the lifetime analysis shows that the UDS group is £1380 more costly and is associated with 0.002 fewer QALYs than the CCA‐only group (Table 3). There is considerable uncertainty relating to the magnitude of both costs and QALYs, with ~50% of samples sitting either side of the *y*‐axis of the cost‐effectiveness plane (Fig. 3), signifying that a QALY gain is as likely as a QALY loss. This is associated with UDS having a 23.4% chance of being cost‐effective at £20 000 per QALY gained, with the full CEAC being shown in Appendix C: Fig. C1.

**Table 3:** Lifetime modelled cost‐effectiveness of UDS and CCA.

	Within trial costs, £	Long‐term costs, £	Total costs, £	Within trial QALYs	Long‐term QALYs	Total QALYs	ICER, £ per QALYs gained	Probability cost‐effective at £20 K per QALY gained, %
Primary analysis								
UDS	3907	33 911	37 818	1.315	9.930	11.245		23.4
CCA	3445	32 993	36 438	1.304	9.943	11.247		76.6
Increment			1380			−0.002	Dominated	
Primary analysis, MUI subgroup								
UDS	3959	34 910	38 869	1.369	10.229	11.598		45.3
CCA	3506	33 802	37 307	1.316	10.223	11.539		54.7
Increment			1562			0.059	26 462	

ICER, incremental cost‐effectiveness ratio.

The model‐based deterministic sensitivity analysis showed that with the shorter time horizon, the probability of UDS being cost‐effective increased to 39.7%. Likewise, using the same utility estimate for all women remaining on treatment, regardless of the specific treatment, increased the probably of cost‐effectiveness to 30.6%. The trial‐based deterministic sensitivity analyses had little impact on the cost‐effectiveness of UDS, except for the complete case analysis for which the probability of cost‐effectiveness of 67.35% (as opposed to 33.8% in the analysis with imputation).

When the MUI subgroup was modelled over the lifetime of women, an alternative parameterisation was adopted using the within trial and 24‐month treatments/utilities for the sub‐group (Appendix D: Table D1). As a consequence of this, the UDS group gained more QALYs than the CCA group, albeit at a higher cost. The incremental cost‐effectiveness ratio for UDS was £26 462, with a probability of being cost‐effective of 45.3% of £20 000 per QALY gained, rising to 53.8% at £30 000 per QALY gained (Appendix C: Figure C1).

## Discussion

### Summary

The primary, model‐based economic analysis showed that UDS has a low probability of being cost effective at £20 000 per QALY gained (23.4%), producing modestly higher costs (£1380) and slightly lower QALYs (−0.002) per patient. This is a more definitive conclusion than that produced by the within‐trial analysis which produced a 33.8% chance of UDS being cost‐effective. The additional certainty of the model‐based finding is generated by the modelling of cost and outcomes beyond the end of the trial, which shows fewer participants receiving BoNT‐A and more women receiving ‘other care’ in the UDS arm; BoNT‐A is associated with a high utility and ‘other care’ is associated with a high cost.

### Strengths and Weakness

The model has several strengths, most notably that its structure aligns with the trial‐based analysis, thereby producing a high level of internal validity (i.e., the costs, QALYs and utilities are based on the same set of patients). It also aligns well with the majority of previous cost‐effectiveness analyses in this disease area. However, we feel that the structure has one important weakness, namely, that the treatment pathways following the choice of initial treatment are very simple. All subsequent treatments, including for example, percutaneous tibial nerve stimulation (PTNS) and urethral bulking, have been bundled up into a single ‘other care’ health state. An amendment to our model structure could accommodate this by allowing transitions to specified therapies after treatment failure, rather than automatically moving to ‘other care’. However, this structural improvement would be completely undermined by the small amount of data relating to those subsequent treatments within this trial and the poor evidence base relating to the long‐term effectiveness of those treatments in this patient population.

The parameterisation of the long‐term component of the model was based on a review of studies reporting the long‐term follow‐up of patients with R‐OAB and of associated economic models. However, that evidence base has several weaknesses. First, the studies are not restricted to UK cohorts and so their results may not reflect UK outcomes. Second, success rates vary considerably between the studies, as do the inclusion/exclusion criteria of the underlying patient populations [16, 17, 18, 19, 20, 21, 22, 23]. Only for the treatment of SUI does the long‐term treatment effectiveness parameters come from a formal systematic review, and even that choice is a simplification as the success rates relate only to the use of mid‐urethral slings as parameters for a mix of treatments relevant to this patient population were not available from the review [19]. Third, whilst the long‐term studies identified included some patients with >10 years of follow‐up, the median follow‐up periods were much shorter, and as such, the observed success rates beyond 5 years are based on relatively small patient numbers (e.g., 55 patients for our SNM estimates in Table 1).

It should also be noted that the estimates of treatment effectiveness beyond 24 months are the same for both initial treatment strategies. As such, the potential benefits of UDS are limited to the initial choice of treatment. However, from a clinical perspective, there is an expectation that if UDS helps with the selection of a more appropriate treatment, that ongoing effectiveness will be greater than for CCA‐only patients, as the treatment will be better aligned to the underlying dysfunction. As such, our estimates may underestimate the cost‐effectiveness of UDS. In the absence of a valid evidence base, a long‐term follow‐up of the patients within FUTURE is underway to help identify such differences.

A summary of the model assumptions and data sources that are considered to be the most important is given in Appendix E: Table E1. Despite these potential problems, it is important to note that the conclusion for the full patient population is the same as that for the within‐trial analysis.

Three issues relating to the underlying trial analysis are also of note. First, the complete case analysis of FUTURE produced a quite different result with UDS plus CCA being shown to be cost‐effective. The reason for this marked divergence from our primary analysis is unclear; however, the appropriateness of multiple imputation was assessed using methods recommended for economic evaluation. Second, several changes to the planned analysis as specified in the Health Economics Analysis Plan (HEAP) were undertaken in relation to the choice of regression methods, the structure of the decision analytical model and the value of information analysis. A full list of changes is given in Appendix F: Table F1. Third, the addition of the 24‐month timepoint for data collection for only some patients, complicates the prediction of costs and QALYs at 24 months as time‐dependence needs to be included within the regression models. Whilst this has been undertaken, there are undoubtedly other ways to specify this time‐dependence, therefore there is some methodological uncertainty associated with the 24‐month cost and QALY estimates.

Finally, the FUTURE study and the economic analysis presented here were designed to reflect UK practice. Consequently, further consideration would be needed when assessing the transferability of the results and conclusions to other healthcare settings. In general terms, our analysis shows the importance of the long‐term effectiveness of any subsequent treatments as captured by the health state utilities and transition probabilities. As such, assessing the relevance of these to other counties would be an important first step before using these results beyond the UK.

### Comparisons with Other Studies

Whilst several studies have assessed the cost‐effectiveness of UDS testing, none relate to the same patient population as the FUTURE study. The study that most closely matches ours is that undertaken by Rachaneni et al. [28], which was based on observational data and concluded that UDS could be cost‐effective in specific patient sub‐groups, although this was limited to a 5‐year time horizon. Likewise, another observational study using data on 199 patients found that clinical assessment (including use of a micturition diary) was equally effective and cheaper than UDS, but this analysis was for a poorly defined patient group and had a limited time horizon [29]. Three other studies have undertaken cost‐effectiveness analyses of UDS, but specifically as a test prior to surgery for SUI [25, 30, 31]. In addition, these studies had several limitations, including limited time horizons, the lack of QALYs and contemporary trial data.

### Further Research

A longer‐term follow‐up for the FUTURE patient cohort is underway and that is expected to reduce the uncertainties relating to several of the issues highlighted above. That study will capture more information on initial post‐assessment treatments and subsequent treatments. It will also help with an assessment of differential success rates for initial treatments between study groups.

## Conclusion

The use of UDS in women with a diagnosis of OAB and who's condition is refractory to initial medical and conservative treatments is unlikely to be cost‐effective when examined from a UK perspective and with a lifetime horizon. Within the trial, this is driven by the costs of UDS itself, whereas the long‐term model suggests that the mix of treatments can produce long‐term differences in patient outcomes and costs. Several problems with the parameterisation of the model were identified and an ongoing long‐term follow‐up study will help reduce these uncertainties.

## Disclosure of Interests

All authors declare a grant (reference number 15/150/05) from the UK National Institute for Health Research Health Technology Assessment Programme (NIHR HTA) was received by University of Aberdeen and Grampian Health Board to undertake the research. Mohamed Abdel‐Fattah declares other financial or non‐financial interests as a speaker, consultant and/or surgical trainer for a number of industrial companies (Astellas, Ethicon, Bard, Pfizer, AMS, Coloplast, and others) with travel expenses reimbursed, and on occasions received personal honorariums and sponsorship towards attending scientific conferences; Research grant from Coloplast managed by University of Aberdeen; Limited number of supported trainees attended pharmaceutical sponsored educational/leadership workshops and/or received assistance towards presenting their research work at scientific conferences; Previous chairman of the Scottish Pelvic Floor Network, which at the time received sponsorship by various industrial companies and fees to exhibit in annual meetings and surgical workshops; Receiving travel sponsorship and occasional speaker fees from numerous national and international conferences and non‐profit organisations when invited as guest speaker and/or expert surgeon; In 2019, at request from NHS Grampian, attended two educational meetings for setting up SNM service partially funded by Medtronic. David Cooper reports grants or contracts from NIHR HTA funding for long‐term follow‐up of the MASTER and SIMS trials. Helen Bell‐Gorrod declares grants or contracts from Merck Sharp & Dohme, NICE and UK Research and Innovation. Simon Dixon reports consulting fees from the NICE, Shionogi and Maverex.

## Supporting information


**Appendix S1.** Health Economics Analysis Plan (HEAP) for the FUTURE study.

## Appendix A

**Table A1:** Full list of resource use items and associated unit costs.

Item of resource use and associated care report form	Cost, £	Source
**Hospital visits**		
Outpatient visit	161.17	NHS Reference costs 2020/2021
Ward review (not admitted)	161.17	NHS Reference costs 2020/2021
Elective hospital admission	2358.92	NHS Reference costs 2020/2021
Emergency hospital admission	509.11	NHS Reference costs 2020/2021
**Investigations**		
Invasive UDS	230.29	NHS Reference costs 2020/2021
Non‐invasive UDS	230.29	NHS Reference costs 2020/2021
Cystoscopy	272.95	NHS Reference costs 2020/2021
MSU test	10.18	NHS Reference costs 2020/2021
Voiding assessment ‐ Catheterisation	213.28	NHS Reference costs 2020/2021
Renal ultrasound scan	64.31	NHS Reference costs 2019/2020
CT	93.94	NHS Reference costs 2019/2020
MRI	325.33	NHS Reference costs 2020/2021
**BoNT‐A treatment sessions** *		
**Drug costs** ^†^		
BoNT‐A 50 unit	71.63	BNF 2021
BoNT‐A 100 unit	166.00	BNF 2021
BoNT‐A 200 unit	268.10	BNF 2021
BoNT‐A 500 unit	308.00	BNF 2021
Cystoscope costs		
Cystoscope + general/regional	731.84	NHS Reference costs 2020/2021
Cystoscope + local/local plus sedation	272.95	NHS Reference costs 2020/2021
**Other medical care and appointments**		
Absorbent Pads^‡^	5.00	NHS price not available. Price based on a pack of 30 pads from on‐line search of products.
Intermittent Catheter	162.12	NHS Reference costs 2020/2021
Medications	Various	NHS Business Services Authority, 2021
Bladder instillation	658.83	NHS Reference costs 2020/2021
Clinic appointment	161.17	NHS Reference costs 2020/2021
Telephone call	119.21	NHS Reference costs 2020/2021
**SNM procedures**		
SNM + permanent + inpatient	9036.45	NHS Reference costs 2020/2021
SNM + permanent + day surgery unit	1614.97	NHS Reference costs 2020/2021
SNM + permanent + main theatre unit	1614.97	NHS Reference costs 2020/2021
SNM + not permanent + inpatient	5429.52	NHS Reference costs 2020/2021
SNM + not permanent + day surgery unit	3540.69	NHS Reference costs 2020/2021
SNM + not permanent + main theatre unit	3540.69	NHS Reference costs 2020/2021
Lead removal	517.30	NHS Reference costs 2020/2021
**SUI procedures**		
Fascial (fascial sling)	7319.10	NHS Reference costs 2020/2021
Urethral bulking agent	321.46	NHS Reference costs 2020/2021
**PTNS**		
Costed via hospital visits.		
**Primary and community care**		
GP	33.00	PSSRU 2021
Practice nurse	21.00	PSSRU 2021
Physiotherapist	20.50	PSSRU 2021
Social care	23.00	PSSRU 2021
**Lost productivity**		
Hourly wage	18.01	Annual Survey of Hours and Earnings 2020/2021

BNF, British National Formulary; CRF, case report form; MSU, mid‐stream urine; PQ, participant questionnaire; PSSRU, Personal Social Services Research Unit.

* BoNT‐A treatments sessions are costed as the sum of two components; drug costs plus the NHS reference cost for cystoscope (general/regional or local plus sedation).

^†^ Costs relate to the mean price across the products that are available for each dose.

^‡^ Provision of incontinence pads by the NHS is not universal across the UK, but for the purposes of our analysis, we made the simplifying assumption that they were an NHS cost.

## Appendix B

**Table E1:** Key modelling features and assumptions.

Assumption	Explanation	Evidence and impact
Bundling all other treatments as ‘other care’ is appropriate	The first line of treatments following randomisation in FUTURE are modelled individually. Successful treatment leads to higher utility and lower costs. ‘Other care’ is modelled as a single health state with constant cost and utility.	Structurally, this is considered to match well with the FUTURE protocol and NICE Guidelines. However, if new treatments become more prominent in this patient group (e.g., PTNS), modelling these individually may be better.
Cost of other care	The cost of other care is based on all care received by patients in the final 12 months of FUTURE, if they did not receive BoNT‐A, SNM or SUI surgery. Whereas the costs of BoNT‐A, SNM and SUI surgery relate only to those specific treatments; as such, any associated care is not included. This could potentially bias the results in favour of the study are that is associated with the most successful treatments.	The size of this potential bias is unknown. Trying to identify ‘background’ care for patients being treated with BoNT‐A, SNM or SUI surgery in FUTURE is problematic. However, given the low rates of BoNT‐A, SNM or SUI surgery within the first 24 months, this is not considered to be a big problem.
Success rates for treatment are the same for both study arms	The absence, or otherwise, of a UDS does not affect the success rates for subsequent treatment. As such, the potential benefits of UDS are limited to the initial choice of treatment. However, from a clinical perspective, there is an expectation that if UDS helps with the selection of a more appropriate treatment, that ongoing effectiveness will be greater than for CCA‐only patients, as the treatment will be better aligned to the underlying dysfunction. As such, our estimates may underestimate the cost‐effectiveness of UDS.	This is incredibly difficult to quantify at this moment in time. FUTURE is by far and away the biggest and most relevant study for examining this issue, but the rates of intervention at 24 months were too low for a robust statistical analysis to be undertaken.
A cohort model based on a single age group is appropriate.	The model is based on the mean values from the FUTURE study, with the mean age of the women being 60 years. As such, the utility estimates from the trial, and the modelled mortality rates will not accurately represent those of women of other ages.	The economic analysis is concerned with the mean *difference* in costs and QALYs between the two study arms, not the absolute levels. If those differences are expected to be the same for all ages, the results are valid if the distribution of ages is the same in both groups. The mean age of the two groups is almost identical. If the impact of UDS is expected to vary by age, undertaking sub‐group analyses for different age groups would be appropriate. However, we do not believe this to be the case.

Data source	Explanation	Evidence and impact
FUTURE trial	The FUTURE trial provides the mean costs and QALYs at 24 months, the treatment designations at 24 months and the annual cost of ‘Other care’. Whilst the study was designed to represent UK practice, it will not accurately represent all patients. Also, the COVID‐19 pandemic caused disruptions to patterns of clinical care and precipitated an extra study questionnaire that necessitated statistical adjustment to produce the 24‐month total.	The design of FUTURE is considered robust; however, we are uncertain about the short‐ and medium‐term impact of COVID‐19 on treatment patterns. The statistical adjustment is considered robust; however, the complete case analysis produced notably different conclusions for the cost‐effectiveness analysis. Overall, our desire to maximise the use of FUTURE and minimise external data sources is considered a strength of our study.
Long‐term success rates for BoNT‐A, SNM or SUI surgery	There are based on a review of studies with the longest follow‐ups. However, these studies are highly variable in terms of country, patient populations and attrition.	These data are central to the long‐term modelling and so there is concern with using these data as their relevance to contemporary UK practice is open to debate. However, we are unaware of any studies that can provide better data. It should be noted that the small sample sizes are factored into the probabilistic sensitivity analysis (as the associated standard errors will be large).
Using mid‐urethral slings (MUS) success rates for all patient receiving SUI surgery	These are taken from a systematic review of all modalities of surgery, but the necessary data can only be derived from the results relating to MUS.	Success rates vary by modality of surgery; however, those for MUS are considered to be the most appropriate as it is the most common procedure (Jha S, Hillard T, Monga A, Duckett J. National BSUG audit of stress urinary incontinence surgery in England. *Int Urogynecol J*. 2019 Aug;30(8):1337–1341)

## Appendix D

**Table B1:** Detailed model inputs.

Parameter	Time point(s), months	Mean	Distribution
**Costs at 24 months (discounted), £**			
UDS + CCA	Up to 24	3907.33	Normal (SE = 466.02)
CCA	Up to 24	3444.78	Normal (SE = 449.79)
**QALYs at 24 months (discounted)**			
UDS + CCA	Up to 24	1.315	Normal (SE = 0.057)
CCA	Up to 24	1.304	Normal (SE = 0.056)
**Last treatment at 24 months (%)**			
UDS + CCA			
BoNT‐A	At 24	49.27	Binomial, 271/550
SNM	At 24	1.82	Binomial, 10/550
SUI surgery	At 24	2.55	Binomial, 14/550
Other	At 24	46.36	Binomial, 255/550
CCA			
BoNT‐A	At 24	61.93	Binomial, 340/549
SNM	At 24	1.09	Binomial, 6/549
SUI surgery	At 24	0.73	Binomial, 4/549
Other	At 24	36.25	Binomial, 199/549
**Utilities at 24 months for**			
BoNT‐A	After 24	0.632	Normal (SE = 0.041)
SNM	After 24	0.599	Normal (SE = 0.036)
SUI surgery	After 24	0.643	Normal (SE = 0.045)
Other	After 24	0.612	Normal (SE = 0.040)
**Unit/Annual costs (undiscounted), £**			
BoNT‐A (applied to re‐treatment)*	After 24	463.75	Deterministic
SNM (applied to revisions)^†^	After 24	1614.97	Deterministic
SUI surgery	After 24	0	Deterministic
Other treatments, e.g., PTNS, urethral bulking^‡^	After 24	1723.31	Normal (SE = 252.79)

* BoNT‐A cost is derived from the Healthcare Resource Group (HRG) LB72A Diagnostic Flexible Cystoscopy, 19 years and over ‐ Outpatient procedure Urology (OPROC) (Appendix D), plus the cost of the mean BoNT‐A dose (120 iu). Re‐treatment happens every 8.2 months.

^†^ SMN revision cost is based on HRG LB79Z Insertion of Neurostimulator Electrodes for Treatment of Urinary Incontinence ‐ day case. Removal is also applied upon transition to ‘Other care’ and is £517.30 LB20G Infection or Mechanical Problems Related to Genito‐Urinary Prostheses, Implants or Grafts, without Interventions, with CC Score 0–1 (Appendix D).

^‡^ Annual cost associated with the ‘other’ health state is estimated as the 24‐month mean cost for those that did not receive BoNT‐A, SNM and SUI from a model with costs associated with the intervention removed, divided by 2 to obtain an annual cost.

## Appendix E

**Table F1:** Deviations from the HEAP.

Deviation from HEAP	HEAP section number	Description and rationale
EQ‐5D‐5L valuation	4.7	The plan was to use the van Hout ‘cross‐walk’ algorithm to value the EQ‐5D‐5L, which is in line with the recommendation of NICE (at the time at which the study protocol was written). Since that time, NICE has changed its recommendation to the use of the Alava‐Hernandez algorithm, and so this was used in a sensitivity analysis
Regression methods for the estimation of costs and QALYs	5.5	The regression models for the within trial analysis was planned to use seemingly unrelated regression, however, the distribution of the data were not appropriate for this approach and so a data‐driven approach based on the modified Park test was adopted. This change is based on good methodological practice (Glick et al. [27]).
Subgroup analysis	5.13	Three subgroup analyses were planned but only the analysis by initial diagnosis (OAB vs MUI) was considered feasible given the small sample sizes available for the other analyses (which were treatment‐based). An analogous change to the statistical analysis plan was undertaken for the same reason
Model structure	6.3	The decision analytical model was to be based on that presented in Rachaneni et al. [28]; however, upon closer examination the data from the FUTURE study were not closely aligned with that model structure, which would have meant discarding a lot of data from FUTURE for those used in Rachaneni et al. [28]. This was considered unsatisfactory and so a conceptual modelling exercise, that included a review of all associated model, and this suggested a more appropriate model structure. That structure maximised the use of FUTURE data and was well aligned with several other related studies
Value of information analysis	6.10	A value of information analysis was planned; however, an extension study was commissioned by the study funders (to make good on disruptions caused by the COVID‐19 pandemic). As such, it was felt that the value of information analysis should be postponed until the extension study was complete

## Appendix F

**Table D1:** The MUI parameter values.

Parameter		Time point(s), months	Mean	Distribution	Source
Costs at 24 months (discounted), £					
UDS + CCA		Up to 24	3958.75	Normal (SE = 525.98)	FUTURE
CCA		Up to 24	3505.61	Normal (SE = 501.84)	FUTURE
QALYs at 24 months (discounted)					
UDS + CCA		Up to 24	1.369	Normal (SE = 0.063)	FUTURE
CCA		Up to 24	1.316	Normal (SE = 0.060)	FUTURE
Last treatment at 24 months, %					
UDS + CCA	BoNT‐A	At 24	43.32	Binomial, 81/187	FUTURE
UDS + CCA	SNM	At 24	2.67	Binomial, 5/187	FUTURE
UDS + CCA	SUI surgery	At 24	3.74	Binomial, 7/187	FUTURE
UDS + CCA	Other	At 24	50.27	Binomial, 94/187	FUTURE
CCA	BoNT‐A	At 24	59.78	Binomial, 110/184	FUTURE
CCA	SNM	At 24	0.54	Binomial, 1/184	FUTURE
CCA	SUI surgery	At 24	2.17	Binomial, 4/184	FUTURE
CCA	Other	At 24	37.50	Binomial, 69/184	FUTURE
Utilities at 24 months for:					
	BoNT‐A	After 24	0.626	Normal (SE = 0.065)	FUTURE
	SNM	After 24	0.569	Normal (SE = 0.065)	FUTURE
	SUI surgery	After 24	0.700	Normal (SE = 0.073)	FUTURE
	Other	After 24	0.637	Normal (SE = 0.065)	FUTURE
Unit/annual costs (undiscounted), £					
	BoNT‐A (applied to re‐treatment)	After 24	463.75	Deterministic	FUTURE
	SNM (applied to revisions)	After 24	1614.97	Deterministic	FUTURE
	SUI surgery	After 24	0	Deterministic	Assumption
	Other	After 24	1765.341	Normal (SE = 275.14)	FUTURE

SE, standard error.
