# Supplementary material for: Long‐term cost‐effectiveness of invasive urodynamic studies for overactive bladder in women
Source: BJU Int. 2025 Apr 19;136(1):82–94. doi: 10.1111/bju.16703 (PMC12134422; doi:10.1111/bju.16703)
Supplement: Supplementary file 1 — Appendix S1. Health Economics Analysis Plan (HEAP) for the FUTURE study. [file BJU-136-82-s001.docx]

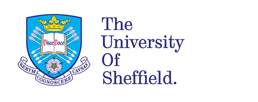

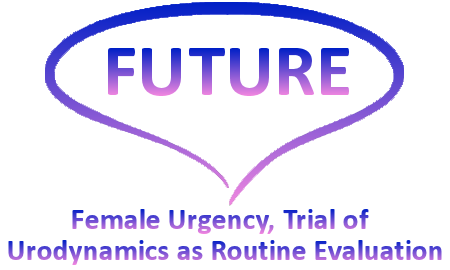

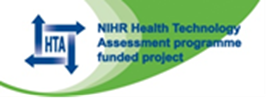


**Health Economics Analysis Plan (HEAP)**

**for the FUTURE Study**

**Authors:**

Professor Simon Dixon ^1^

Dr Helen Bell Gorrod ^1^

Professor Mohammed Abdel-Fattah ^2^

1. Health Economics and Decision Science (HEDS), School of Health and Related Research (ScHARR), University of Sheffield. Regent Court, 30 Regent Street, Sheffield, S1 4DA
2. Aberdeen Centre for Women Health Research, Aberdeen Maternity Hospital, Foresterhill, Aberdeen, AB25 ZH

Correspondence to: [s.dixon@sheffield.ac.uk](mailto:s.dixon@sheffield.ac.uk)

**Trial HEAP version:** 2.1; Date: 6/12/2022

Table of Contents

[1. Section 1: Administrative Information 4](#_Toc67042870)

[**1.1** **Study Title** 4](#_Toc67042871)

[**1.2** **Trial registration number** 4](#_Toc67042872)

[**1.3** **Source of funding** 4](#_Toc67042873)

[**1.4** **Purpose of HEAP** 4](#_Toc67042874)

[**1.5** **Trial protocol version** 4](#_Toc67042875)

[**1.6** **Trial SAP version** 4](#_Toc67042876)

[**1.7** **Trial HEAP version** 4](#_Toc67042877)

[**1.8** **Trial HEAP Revisions** 5](#_Toc67042878)

[**1.9** **Roles and Responsibilities** 7](#_Toc67042879)

[**1.10** **Signatures** 7](#_Toc67042880)

[**1.11** **Acronyms and abbreviations** 8](#_Toc67042881)

[2. Section 1: Trial Introduction and Background 9](#_Toc67042882)

[**2.1** **Trial background and rationale** 9](#_Toc67042883)

[**2.2** **Aim of the trial** 9](#_Toc67042884)

[**2.3** **Objectives and research hypothesis of the trial** 9](#_Toc67042885)

[**2.4** **The trial population** 9](#_Toc67042886)

[**2.5** **Intervention and comparator** 10](#_Toc67042887)

[**2.6** **Trial design** 10](#_Toc67042888)

[**2.7** **Trial start and end date** 10](#_Toc67042889)

[3. Economic Evaluation Overview 10](#_Toc67042890)

[**3.1** **Aim of the economic evaluation** 10](#_Toc67042891)

[**3.2** **Objectives of the economic evaluation** 11](#_Toc67042892)

[**3.3** **Overview of economic analysis** 11](#_Toc67042893)

[**3.4** **Jurisdiction** 11](#_Toc67042894)

[**3.5** **Perspective** 11](#_Toc67042895)

[**3.6** **Time horizons** 11](#_Toc67042896)

[4. Economic Data Collection and Management 11](#_Toc67042897)

[**4.1** **Statistical software** 11](#_Toc67042898)

[**4.2** **Identification of resource use** 11](#_Toc67042899)

[**4.3** **Measurement of resource use** 11](#_Toc67042900)

[**4.4** **Valuation of resource use** 12](#_Toc67042901)

[**4.5** **Identification of outcomes** 12](#_Toc67042902)

[**4.6** **Measurement of outcomes** 12](#_Toc67042903)

[**4.7** **Valuation of outcomes** 12](#_Toc67042904)

[5. Economic Data Analysis 12](#_Toc67042905)

[**5.1** **Analysis population** 12](#_Toc67042906)

[**5.2** **Timing of the analysis** 13](#_Toc67042907)

[**5.3** **Discounting rates for costs and benefits** 13](#_Toc67042908)

[**5.4** **Cost-effectiveness threshold** 13](#_Toc67042909)

[**5.5** **Statistical decision rules** 13](#_Toc67042910)

[**5.6** **Analysis of resource use** 13](#_Toc67042911)

[**5.7** **Analysis of costs** 13](#_Toc67042912)

[**5.8** **Analysis of outcomes** 13](#_Toc67042913)

[**5.9** **Data cleaning for analysis** 13](#_Toc67042914)

[**5.10** **Missing data** 13](#_Toc67042915)

[**5.11** **Analysis of cost-effectiveness** 13](#_Toc67042916)

[**5.12** **Addressing uncertainty** 14](#_Toc67042917)

[**5.13** **Subgroup analysis** 14](#_Toc67042918)

[**5.14** **Sensitivity analysis** 14](#_Toc67042919)

[6. Modelling 15](#_Toc67042920)

[**6.1** **Decision-analytic modelling** 15](#_Toc67042921)

[**6.2** **Model type** 15](#_Toc67042922)

[**6.3** **Model structure** 15](#_Toc67042923)

[**6.4** **Treatment effect beyond the end of the trial** 15](#_Toc67042924)

[**6.5** **Methods for identifying and estimating parameters** 15](#_Toc67042925)

[**6.6** **Deviations from the pre-existing model** 16](#_Toc67042926)

[**6.7** **Model uncertainty** 16](#_Toc67042927)

[**6.8** **Model validation** 16](#_Toc67042928)

[**6.9** **Subgroup analysis** 16](#_Toc67042929)

[7. Reporting/Publishing 16](#_Toc67042930)

[**7.1** **Decision-analytic modelling** 16](#_Toc67042931)

[**7.2** **Deviations from the HEAP** 16](#_Toc67042932)

[8. References 16](#_Toc67042933)

# Section 1: Administrative Information

- 1. **Study Title**

FUTURE Study - Female Urgency, Trial of Urodynamics as Routine Evaluation: a superiority randomised clinical trial to evaluate the effectiveness and cost effectiveness of invasive urodynamic investigations in management of women with refractory overactive bladder symptoms

- 1. **Trial registration number**

ISRCTN: 63268739

- 1. **Source of funding**

National Institute for Health Research (NIHR) Health Technology Assessment Programme; Reference number - HTA 15/150/05

- 1. **Purpose of HEAP**

The purpose of this HEAP is to describe the analysis and reporting procedure intended for economic analysis to be undertaken. The analysis plan is designed to ensure that there is no conflict with the protocol and associated statistical analysis plan (SAP) and it should be read in conjunction with them.

- 1. **Trial protocol version**

This Document has been written based on information contained in the trial protocol version 5.0, dated 01/05/2019

- 1. **Trial SAP version**

Version Final, dated 15/11/2022

- 1. **Trial HEAP version**

Version 2.1; dated 6/12/2022

- 1. **Trial HEAP Revisions**

| Updated HEAP version No. | Protocol version | Section number changed | Description of, and reason for, change | Individual making the change | Date changed |
| --- | --- | --- | --- | --- | --- |
| 0.1 | 5.0 | - | First draft | AA | 13/02/2020 |
| 1.0 | 5.0 | Various sections | Document updated based on comments from the lead health economist, chief investigator and trial statisticians | AA | 19/03/2021 |
| 2.0 | 5.0 | Title page | Change in authorship and corresponding author | SD | 15/11/22 |
|  |  | 1.9 | Change in roles and responsibilities in line with the title page. | SD | 15/11/22 |
|  |  | 2.7, 3.6 and 6.8 | Recognition of the addition of a 24 month follow-up from some patients and the necessary extension of the time horizon of the analysis. | SD | 15/11/22  6/12/22 |
|  |  | 4.1 | Change of software options for the long-term modelling. R has been replaced by Treeage. | SD | 15/11/22 |
|  |  | 4.3 and 4.7 | The additional data collection point of 24 months has been included. | SD | 15/11/22 |
|  |  | 4.7 | The estimation of utility decrement associated with urodynamics has been changed so that it is in line with the study protocol. | SD | 15/11/22 |
|  |  | 5.5 | Title of section changed from “Statistical decision rules” to “Statistical model” to better reflect its content. | SD | 15/11/22 |
|  |  | 5.13 | The wording of the sub-group analysis is changed to match that in the SAP. | SD | 6/12/22 |
|  |  | 6.2 | The tying the model development to that of the previous HTA proposal has been dropped. Whilst aspects of this are useful, it is misleading to say that our planned analysis will be “adapted” from that model. | SD | 15/11/22 |
|  |  | 6.3 | The description of the sub-group analysis had been deleted to avoid duplication (as it is described again, more appropriately, in Section 6.9). |  | 6/12/22 |
|  |  | 6.10 | The specification of SAVI for the EVI analysis has been removed as other platforms are equally valid. |  | 15/11/22 |

- 1. **Roles and Responsibilities**

The HEAP was prepared by Mr Abualbishr Alshreef (Health Economist) and approved by, Prof Simon Dixon (Lead Health Economist) and Prof Mohammed Abdel-Fattah (Chief Investigator). The trial health economists are responsible for conducting and reporting the economic evaluation in accordance with the HEAP.

**Lead Health Economist:**

**Name:** Prof Simon Dixon

**Address:** Health Economics and Decision Science (HEDS), School of Health and Related Research (ScHARR), University of Sheffield, Regent Court, 30 Regent St, Sheffield, S1 4DA

**Email:** [s.dixon@sheffield.ac.uk](mailto:s.dixon@sheffield.ac.uk)

**Phone:** 0114 222 0724

**Health Economist:**

**Name:** Dr Helen Bell Gorrod

**Address:** Health Economics and Decision Science (HEDS), School of Health and Related Research (ScHARR), University of Sheffield, Regent Court, 30 Regent St, Sheffield, S1 4DA

**Email:** [helen.bellgorrod@sheffield.ac.uk](mailto:helen.bellgorrod@sheffield.ac.uk)

**Phone:** 0114 222 0890

**Chief Investigator:**

**Name**: Prof Mohamed Abdel-Fattah

**Address**: Aberdeen Centre For Women Health Research, Aberdeen Maternity Hospital, Foresterhill, Aberdeen, AB25 ZH

**Email**: [m.abdelfattah@abdn.ac.uk](mailto:m.abdelfattah@abdn.ac.uk)

**Phone:** 0122 222 8424

- 1. **Signatures**

Lead Health Economist:

Prof Simon Dixon

Signature
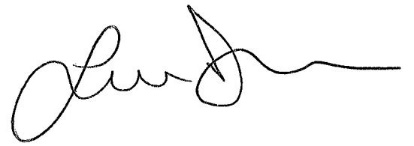
 Date 6^th^ December 2022

Health Economist

Dr Helen Bell Gorrod

Signature Date

Chief Investigator

Prof Mohamed Abel-Fattah

Signature Date

- 1. **Acronyms and abbreviations**

| BoNT-A | Botulinum Toxin injection |
| --- | --- |
| BNF | British National Formulary |
| CUA | Cost Utility Analysis |
| CEAC | Cost Effectiveness Acceptability Curve |
| CHEERS | Consolidated Health Economic Evaluation Reporting Standards |
| DSA | Deterministic Sensitivity Analysis |
| GP | General Practitioner |
| HTA | Health Technology Assessment |
| HRQL | Health-Related Quality of Life |
| HEAP | Health Economic Analysis Plan |
| HED | Health Economics and Decision Science |
| HEAP | Health Economic Analysis Plan |
| ITT | Intention To Treat |
| ICER | Incremental Cost Effectiveness Ratio |
| MUI | Mixed Urinary Incontinence |
| NHS | National Health Service |
| NIHR | National Institute for Health and Care Excellence |
| OAB | Overactive Bladder |
| PSSRU | Personal Social Service Research Unit |
| PSA | Probabilistic Sensitivity Analysis |
| QALY | Quality-Adjusted Life Years |
| ScHARR | School of Health and Related Research |
| SAP | Statistical Analysis Plan |
| SUI | Stress Urinary Incontinence |
| SNM | Sacral Neuro-Modulation |
| SUR | Seemingly Unrelated Regression |
| SAVI | Sheffield Accelerated Value of Information |
| UDS | Urodynamics Study |

# Section 1: Trial Introduction and Background

- 1. **Trial background and rationale**

Overactive bladder (OAB) is the symptom of urinary urgency and is often associated with frequency, Nocturia and urgency urinary incontinence.^1^ This problem has a negative impact on woman’s social, physical and psychological wellbeing; and significant cost implications to the health resources in the UK. The total annual cost of management of urinary incontinence (UI) to the UK National Health Service (NHS) is estimated £301 million or 0.3% of the total NHS budget in 2009.^2^

Evidence generated from the FUTURE study would inform decision-making on whether routine Urodynamics investigation improves the treatment outcomes in women with refractory OAB and whether it is cost-effective.

This Health Economics Analysis Plan (HEAP) outlines the planned procedure for conducting the Health Economic Evaluation sub-study of the FUTURE project.

- 1. **Aim of the trial**

The FUTURE study is a pragmatic multicentre superiority randomised controlled trial comparing the effectiveness and cost-effectiveness of routine Urodynamics investigation and comprehensive clinical assessment versus comprehensive clinical assessment only in the management of women with refractory OAB symptoms

- 1. **Objectives and research hypothesis of the trial**

**Hypothesis:**

In women with refractory OAB, Urodynamics and comprehensive clinical assessment is associated with superior patient reported outcomes following treatment and is more cost-effective, compared to comprehensive clinical assessment alone.

**Primary Objectives**:

1. Evaluate whether routine Urodynamics investigation and comprehensive clinical assessment significantly improves patient reported success rates following treatment, compared to comprehensive clinical assessment only;
2. Assess the cost-effectiveness of routine Urodynamics investigation and comprehensive clinical assessment, compared to comprehensive clinical assessment only.

**Secondary objectives:**

1. Assess the above outcomes in pre-specified subgroups of women: OAB and urgency predominant mixed urinary incontinence (MUI);
2. Explore the clinicians’ attitude towards Urodynamics investigation and its influence on surgical decision-making;
3. Explore the participants' attitudes and experience in both pathways;
4. Explore the clinical and cost-effectiveness of different sequence of treatments for refractory OAB.
   1. **The trial population**

The target population, to which inferences from the end of this trial are intended to generalise, is the population of women aged ≥18 years, with refractory OAB symptoms.

Inclusion Criteria: Eligible women are women with refractory OAB or urgency predominant MUI in whom OAB are their most bothersome symptoms, and:

- have failed conservative (as per NICE guideline e.g. pelvic floor muscle training/ bladder retraining) and
- have failed or have not tolerated pharmacological treatment (at least 2 different drugs) unless contra-indicated and
- are being considered for further treatment.

Exclusion Criteria:

- Predominant SUI symptoms;
- Previous Urodynamics in the last 12 month;
- Pelvic malignancy or clinically significant pelvic mass;
- Bladder Pain Syndrome;
- Neurological disease (e.g. Parkinson’s disease, spinal injuries, etc);
- Urogenital fistulae;
- Previous treatment with Botulinum Toxin injection (BoNT-A)/ Sacral Neuro-Modulation (SNM) for urinary incontinence;
- Previous pelvic radiotherapy;
- Prolapse beyond introitus;
- Pregnant or planning pregnancy;
- Recurrent UTI where a significant pathology has not been excluded;
- Inability to give an informed consent.
  1. **Intervention and comparator**

Intervention: Urodynamics and comprehensive clinical assessment

Comparator: Comprehensive clinical assessment only.

- 1. **Trial design**

The FUTURE study is pragmatic, multicentre, superiority, randomised controlled trial, which aims to demonstrate a minimum of 10% superiority. For 90% power and a 5% level of significance, 986 participants (493/ group) are needed, rising to 1096 (or 548/group) to allow for 10% attrition at 15 months post randomisation. Study participants are being recruited from 61 secondary and tertiary care hospitals across the UK.

- 1. **Trial start and end date**

Recruitment started in October 2017 and was due to finish in May 2020. The follow-up period was to run for 15 months until August 2021. The impact of COVID 19 pandemic on research in the UK meant that both recruitment and follow-up periods were extended.  In addition, a 24-month follow-up questionnaire/medical note review was included for women who had their treatments delayed (see Study Protocol for details).

# Economic Evaluation Overview

- 1. **Aim of the economic evaluation**

The aim of the economic evaluation is to assess the cost-effectiveness of Urodynamics investigation and comprehensive clinical assessment, compared to comprehensive clinical assessment only.

- 1. **Objectives of the economic evaluation**

The primary objective is to estimate the long-term cost-effectiveness of “Urodynamics and comprehensive clinical assessment” versus “comprehensive clinical assessment only” in the treatment of women with refractory OAB symptoms using economic modelling techniques. A secondary objective is to estimate the short-term cost-effectiveness in a within-trial cost-utility analysis over the trial follow-up of 15 months.

- 1. **Overview of economic analysis**

A cost-utility analysis (CUA) will be used to conduct the economic evaluation. The within trial cost-utility analysis will be performed using individual patient-level data from the FUTURE study. The long-term model-based CUA analysis will be performed using data from the FUTURE study supplemented with external evidence.

- 1. **Jurisdiction**

The trial is conducted in the UK NHS context, which is a publicly funded healthcare system, primarily free at the point of use.

- 1. **Perspective**

The economic evaluation will take the NHS and Personal Social Service perspective. A societal perspective will be applied in a sensitivity analysis.

- 1. **Time horizons**

The primary model-based economic analysis will use the patient lifetime timeframe. The secondary within trial analysis will use a time horizon aligned to the trial. This was originally planned as 15 months, but due to the impact of the COVID-19 pandemic, patients can either have 15 or 24 months of follow-up data. A 24 month time horizon will be used, with patients having a shorter follow-up, having their costs and QALYs modelled statistically.

# Economic Data Collection and Management

- 1. **Statistical software**

Stata MP/4 version 16 (or higher) will be used for data analysis including the within-trial cost-utility analysis. Microsoft Excel or TreeAge will be used for building the long-term economic model.

- 1. **Identification of resource use**

The following items of resource use that may differ between arms will be measured: interventions, hospital contacts, contact with NHS healthcare professionals (GP, practice nurses, physiotherapist/continence nurse, others), contact with private healthcare professionals, over the counter medications, personal social care, further medical care (absorbent pads, catheters, medications) and productivity losses.

- 1. **Measurement of resource use**

Patient-level data are collected for the study interventions: Urodynamics and comprehensive clinical assessment versus comprehensive clinical assessment only, plus subsequent treatments, investigations and other health service contacts. Data are collected at 6 and 15 months post-randomisation (3 months and 12-month post-treatment) via a review of patient medical records. Data collection at 24 months has been undertaken for those women who’s treatment has been significantly delayed by the COVID-19 pandemic. These data are entered onto the study database at these points.

Primary care contacts are collected via patient questionnaires. Resource use data on the costs to patients of undergoing treatment and personal expenditure on products relating to their OAB symptoms are captured by a questionnaire at baseline, 6 ,15 and 24 months (the latter is when applicable).

In addition, data on wider societal costs are collected from participants. Such costs include time taken away from normal activities due to treatment or symptoms, including time taken off work due to treatment of symptoms. Reduced productivity at work due to symptoms are also recorded. These data are collected using the resource use questionnaires administered at baseline, 6, 15 and 24 months.

- 1. **Valuation of resource use**

Each of these resource use items will be allocated a unit cost using the following standard health economic sources:

1. British National Formulary (BNF); ^3^
2. NHS Reference Costs; ^4^
3. The Personal Social Services Research Unit (PSSRU) Unit Costs of Health and Social Care.^5^

The references for each of these unit cost sources are provided for information. The most up-to-date version of each source at the point of analysis will be used.

- 1. **Identification of outcomes**

The primary outcome measure for the economic evaluation will be Quality-Adjusted Life Years (QALY) derived from utility scores obtained using the EQ-5D-5L instrument.

- 1. **Measurement of outcomes**

Health-related Quality of Life (HRQL) will be measured at baseline; at the intervention stage (i.e. Urodynamics); and at 6 , 15 and 24 months post randomisation using the EQ-5D-5L questionnaire.^6^

- 1. **Valuation of outcomes**

QALYs will be calculated using EQ-5D-5L utility scores mapped onto the UK EQ-5D-3L valuation set using the van Hout crosswalk mapping function.^7, 8^ QALYs will be estimated using linear interpolation between the baseline, 6 month, 15 month and 24 month time points.

QALYs will be adjusted to take account of the disutility from Urodynamics (e.g. anxiety and discomfort) based on an exploratory analysis of EQ-5D-5L data from the trial. This analysis will take the form of a regression using the difference between post-urodynamics and pre-urodynamics utility as the dependent variable, and time since urodynamics, its squared term, and other covariates as the independent variables. The resultant disutility associated with urodynamics will be used as an adjustment to the QALYs of all women undergoing urodynamics if p<0.1 on either of the two time covariates.

# Economic Data Analysis

- 1. **Analysis population**

The economic analysis will be based on “intention to treat” (ITT) strategy including all randomised participants.

- 1. **Timing of the analysis**

The economic analysis will be performed once all patients have been followed for 15 months (and 24 months when applicable) after randomisation. The analysis will include a within-trial analysis taking 15 months’ time horizon (secondary analysis), and a model-based analysis extrapolating beyond the end of the trial for a patient lifetime (primary analysis). No interim economic analysis is planned.

- 1. **Discounting rates for costs and benefits**

Both cost and QALYs will be calculated up to 15-months follow-up period and will be discounted at 3.5% p.a. as recommended by NICE.^9^

- 1. **Cost-effectiveness threshold**

As recommended by NICE, a cost-effectiveness threshold of £20,000-30,000 per QALY will be used to make a judgement about cost-effectiveness.^9^

- 1. **Statistical model**

Mean differences in costs and QALYs (incremental) will be estimated alongside their 95% confidence intervals. A Seemingly Unrelated Regression (SUR) model will be used to estimate incremental costs and QALYs. The SUR model will be used to adjust for any imbalance in baseline utility.

- 1. **Analysis of resource use**

Differences in resource use between randomised groups will be described, but not be compared statistically.

- 1. **Analysis of costs**

Mean costs (total costs and disaggregated costs) between randomised groups will be calculated and reported descriptively alongside their 95% confidence intervals, but not be compared statistically.

- 1. **Analysis of outcomes**

The mean EQ-5D utility scores at each follow-up time point will be estimated for each arm. These will be described in tables and graphs alongside their 95% confidence intervals.

- 1. **Data cleaning for analysis**

The raw data will be cleaned using face validity tests and corrected for errors. All corrections will be documented and discussed with the study team before running the final analysis.

- 1. **Missing data**

The data will be checked for missing values and the appropriate methods will be followed to deal with missing data using published guidelines.^10^ The primary analysis will be based on imputation to account for missing data, with additional sensitivity analysis using complete cases. Depending on the patterns of missing data, multiple imputation techniques may be used. Baseline covariates (age, diagnosis of overactive bladder) will be included in the imputation model for imputing missing data using multiple imputation.

- 1. **Analysis of cost-effectiveness**

A cost-utility analysis will be performed where the results will be expressed in terms of cost per QALY gained. The primary model-based analysis and secondary within-trial analysis are described in more details in the following subsections.

The estimated mean total QALYs and costs associated with each treatment option will be used to calculate the Incremental Cost Effectiveness Threshold (ICER). An ICER will be calculated using the following formula:

$$ICER=\frac{cost per patient in intervention arm - cost per patient in control arm}{QALYs per patient in intervention arm - QALYs per patient in control arm}$$

A cost-effectiveness threshold of £20,000 per QALY will be used to judge whether the intervention is cost-effective or not, although the upper bound threshold of £30,000 per QALY will be considered in a sensitivity analysis. For addressing uncertainty, a range of threshold values ranging from £0 to 100,000 will be used for estimating the probability of cost-effectiveness.

The within-trial analysis will be conducted in line with the recommendations by Ramsey et al. (2015) for cost-effectiveness analysis alongside clinical trials.^11^ Specifically, the analysis will use unit costs consistent with measured resource use, using EQ-5D-5L responses mapped into EQ-5D-3L utilities as the measures of health outcome, and follow the guiding principles outlined for the analysis of economic measures. The baseline analysis will be based on imputation to account for missing data, with additional sensitivity analysis using complete cases. The analysis will calculate total costs and quality adjusted life years (QALYs) for each patient and estimate the incremental costs and QALYs using the SUR model.

- 1. **Addressing uncertainty**

In the within-trial analysis, a parametric approach will be used to address uncertainty based on the output from the SUR regression. Particularly, five parameters from the SUR regression output (incremental costs, standard error of the difference in costs, incremental QALYs, standard error of the difference in QALYs, and covariance between cost and QALYs) will be used to generate the CEAC. Bootstrapping will be performed to generate the cost-effectiveness plane.

- 1. **Subgroup analysis**

The economic analysis will explore the following pre-specified subgroup of participants with OAB vs urgency predominant MUI:

- There will be a comparison between participants with OAB and MUI.
- There will also be a comparison between the clinical effectiveness of the different treatment pathways of those who started on BoNT-A and those who start with SNM treatment.
- There will also be a comparison of the effectiveness of (1 SNM and (2) BoNT-A according to clinical assessment compared to treatment which was guided by urodynamics.
  1. **Sensitivity analysis**

In the within-trial analysis, the following sensitivity analyses will be performed.

- Complete case analysis.
- Societal perspective that includes patient costs and production losses.
- Alternative methods for incorporating disutility associated with Urodynamic assessment
  - Replacing negative disutilities (i.e. when utility on the day of assessment is higher than baseline utility) with zero (i.e. no disutility).
  - Assuming disutility lasts for one month and three months (as opposed to one week in the baseline analysis).
- Use of a £30,000 cost-effectiveness threshold.

Further sensitivity analyses may be undertaken if unanticipated problems with the economic data are encountered. These will be reported separately as “unplanned sensitivity analyses”.

# Modelling

- 1. **Decision-analytic modelling**

The primary analysis is model-based. Such an approach is considered superior to trial-based analyses as it can be designed to better fit the research question and incorporate other relevant sources of data. In this particular situation, the model will incorporate the longer-term costs and consequence of using Urodynamics which cannot be observed in the trial. However, the first 15 months (and 24 when applicable) of the model will be based on the trial results.

- 1. **Model type**

A long-term decision tree model will be developed to describe the options being compared and their treatment pathways

- 1. **Model structure**

The structure of the model beyond 15 months will be based on a pre-existing model^41^. A key improvement over this model is the use of more appropriate utilities taken from the trial. In addition, targeted literature searches are undertaken to assess whether any relevant new studies have been published for the other parameters. Improvements to the model design/structure will be considered and changes will be discussed with clinicians before implementation.

- 1. **Treatment effect beyond the end of the trial**

Persistence of treatment effect beyond the trial follow-up will be assessed using standard extrapolation methods. This will be based around model structure and data used to derive the transition probabilities.

- 1. **Methods for identifying and estimating parameters**

Data on the clinical effectiveness of the intervention vs comparator will be obtained from the FUTURE trial for estimating the model parameters. These will include probabilities alongside their 95% confidence intervals, proportions of patients going via different pathways in the model, costs and utilities. Appropriate distributions will be assigned to each parameter value for the probabilistic analysis.

For some parameters of interest where data was not collected in the FUTURE trial (or longer-term estimates), the values for these will be obtained from the Rachaneni model.^12^ Where necessary, these parameter values will be updated using targeted literature searches.

- 1. **Deviations from the pre-existing model**

All assumptions and data sources used in the pre-existing model will be reviewed and changed where appropriate. All such changes (except for the use of FUTURE trial data) will be identified as “Unplanned changes to model parameters”.

- 1. **Model uncertainty**

In the model-based analysis, a probabilistic sensitivity analysis is undertaken on the results and its associated ICER, cost effectiveness plane and CEACs will be generated. Deterministic sensitivity analyses will be undertaken to look at the three sources of methodological uncertainty; societal perspective and the length of disutility associated with Urodynamics

- 1. **Model validation**

24-month scenario analysis in the model should give results similar to the within-trial analysis. This will be used to assess the model validity for estimating the short-term costs and QALYs. To validate extrapolation, we will compare the model results with those from similar studies.

- 1. **Subgroup analysis**

The model-based subgroup analysis will match those specified for the within-trial analysis (Section 5.13)

- 1. **Value of information analysis**

Value of information analysis will be undertaken. The partial values will be used to identify those parameters where there is the greatest value in resolving outstanding uncertainty.

# Reporting/Publishing

- 1. **Decision-analytic modelling**

The Consolidated Health Economic Evaluation Reporting Standards (CHEERS) will be followed when reporting the health economic evaluation. It is anticipated that the followed outputs will be published form the economic analysis.

1. A report of methods and results to constitute a chapter of the HTA monograph
2. An outline of the cost-effectiveness methods and results in the primary paper
3. A peer-reviewed journal paper reporting on the cost effectiveness results
4. A conference abstract
   1. **Deviations from the HEAP**

Any deviations from the HEAP will be described and justified in the final report

# References

1. Abrams P, Cardozo L, Fall M, et al. The Standardisation of Terminology in Lower Urinary Tract Function: Report From the Standardisation Sub-Committee of the International Continence Society. *Urology*. 2003;61(1):37-49. doi:10.1016/s0090-4295(02)02243-4

2. Turner DA, Shaw C, McGrother CW, Dallosso HM, Cooper NJ. The Cost of Clinically Significant Urinary Storage Symptoms for Community Dwelling Adults in the UK. *BJU Int*. 2004;93(9):1246-1252. doi:10.1111/j.1464-410x.2004.04806.x

3. National Institute for Health and Care Excellence. British National Formulary (BNF). NICE. Accessed 14 February, 2020. <https://bnf.nice.org.uk/>

4. Department of Health and Social Care. Reference Costs 2017/18. Accessed 14 February, 2020. <https://improvement.nhs.uk/resources/reference-costs>

5. Curtis LA, Burns A. *Unit Costs of Health and Social Care 2019*. 2019:1-176.

6. Herdman M, Gudex C, Lloyd A, et al. Development and Preliminary Testing of the New Five-Level Version of EQ-5D (EQ-5D-5L). *Qual Life Res*. 2011;20(10):1727-1736. doi:10.1007/s11136-011-9903-x

7. Hout Bv, Janssen M, Feng Y, et al. Interim scoring for the EQ-5D-5L: mapping the EQ-5D-5L to EQ-5D-3L value sets. *Value Health* 2012;15(5):708-715.

8. National Institute for Health and Care Excellence. Position statement on use of the EQ-5D-5L valuation set. NICE. Accessed 13 February, 2020. <https://www.nice.org.uk/Media/Default/About/what-we-do/NICE-guidance/NICE-technology-appraisal-guidance/eq5d5l_nice_position_statement.pdf>

9. National Institute for Health and Care Excellence. Guide to the Methods of Technology Appraisal. NICE. Accessed 14 February, 2020. <https://www.nice.org.uk/process/pmg9/resources/guide-to-the-methods-of-technology-appraisal-2013-pdf-2007975843781>

10. Faria R, Gomes M, Epstein D, White IR. A Guide to Handling Missing Data in Cost-Effectiveness Analysis Conducted Within Randomised Controlled Trials. *PharmacoEconomics*. 2014;32(12):1157-1170. doi:10.1007/s40273-014-0193-3

11. Ramsey SD, Willke RJ, Glick H, et al. Cost-Effectiveness Analysis Alongside Clinical Trials II: An ISPOR Good Research Practices Task Force Report. *Value in Health* 2015;18(2):161-172. doi:10.1016/j.jval.2015.02.001
